# *ZNF300* Knockdown Inhibits Forced Megakaryocytic Differentiation by Phorbol and Erythrocytic Differentiation by Arabinofuranosyl Cytidine in K562 Cells

**DOI:** 10.1371/journal.pone.0114768

**Published:** 2014-12-08

**Authors:** Jinyang Cai, Rui Gong, Fengjuan Yan, Chunjie Yu, Lu Liu, Wei Wang, Yi Lin, Mingxiong Guo, Wenxin Li, Zan Huang

**Affiliations:** 1 State Key Laboratory of Virology, Wuhan University, Wuhan, Hubei, China; 2 College of Life Sciences, Wuhan University, Wuhan, Hubei, China; 3 Hubei International Travel Healthcare Center, Hubei Entry-Exit Inspection and Quarantine Bureau of P. R. China, Wuhan, Hubei, China; University of Hawaii Cancer Center, United States of America

## Abstract

Previously, we reported that *ZNF300* might play a role in leukemogenesis. In this study, we further investigated the function of ZNF300 in K562 cells undergoing differentiation. We found that ZNF300 upregulation in K562 cells coincided with megakaryocytic differentiation induced by phorbol-12-myristate-13-acetate (PMA) or erythrocytic differentiation induced by cytosine arabinoside (Ara-C), respectively. To further test whether ZNF300 upregulation promoted differentiation, we knocked down *ZNF300* and found that *ZNF300* knockdown effectively abolished PMA-induced megakaryocytic differentiation, evidenced by decreased CD61 expression. Furthermore, Ara-C-induced erythrocytic differentiation was also suppressed in *ZNF300* knockdown cells with decreased γ-globin expression and CD235a expression. These observations suggest that ZNF300 may be a critical factor controlling distinct aspects of K562 cells. Indeed, *ZNF300* knockdown led to increased cell proliferation. Consistently, *ZNF300* knockdown cells exhibited an increased percentage of cells at S phase accompanied by decreased percentage of cells at G0/G1 and G2/M phase. Increased cell proliferation was further supported by the increased expression of cell proliferation marker PCNA and the decreased expression of cell cycle regulator p15 and p27. In addition, MAPK/ERK signaling was significantly suppressed by *ZNF300* knockdown. These findings suggest a potential mechanism by which *ZNF300* knockdown may impair megakaryocytic and erythrocytic differentiation.

## Introduction

Krüppel-associated box (KRAB)-containing zinc finger proteins (ZFPs) comprise a large family of transcription regulators in mammals. KRAB-ZFPs typically bear an N-terminal KRAB (Krüppel-associated box) domain that functions to suppress transcription by recruiting KRAB domain-associated protein 1 (KAP-1). KAP1 subsequently recruits histone deacetylase and histone methyltransferase machinery to mediate heterochromatinization and gene silencing [1]–[7]. Based on the structure of the KRAB domain, the KRAB-ZFPs can be further classified into three subfamilies: KRAB (AB) with a classical A-box and a B-box, KRAB (A) with a classical A-box only, and KRAB (Ab) with a classical A-box and a highly divergent B-box [8]. The A-box is highly conserved and plays a key role in the repression of target genes while the B-box is less conserved and plays an auxiliary role [9]. It's been reported that the KRAB-ZFPs are only found in the tetrapod vertebrate, suggesting an important function of KRAB-ZFPs in the evolution process of the higher organisms [10], [11].

ZNF300 is a typical member of KRAB-ZNFs. It was originally isolated from the human embryos based on the enrichment of C2H2-specific mRNA and primarily expressed in heart, skeletal muscle, and brain. It encodes a KRAB domain and 12 C2H2 type zinc finger domains as a nuclear protein. The KRAB domain of the ZNF300 protein exhibits typical transcription repressor activity [12] while the zinc finger domain binds the consensus sequence C(t/a)GGGGG(g/c)G that are found in the promoter regions of multiple genes such as *IL2*, *IL2RB*, *CD44*, *TP53*, tumor necrosis factor-α (*TNF*α), and TNF-α receptor associated factor 2 (*TRAF2*) [13]. Indeed, ZNF300 was shown to activate IL-2Rβ promoter activity[13]. Recently, inflammation was shown to upregulate *ZNF300* expression, which further increased NF-κB activity by up-regulating *TRAF2* and interacting with IKKβ [14]. *ZNF300* upregulation also induced the expression of *IL6* and *IL8*, which may lead to the exacerbation of inflammation and tumor metastasis [14]. In addition, *ZNF300* was downregulated during embryonic stem cell differentiation *in vitro* [15] and associated with 5q-syndrome, a distinct subtype of primary myelodysplastic syndrome (MDS) defined by interstitial deletion of chromosome 5q31-33 [16], [17]. Our previous studies also showed that *ZNF300* was associated with myeloid differentiation [18]. Although these data suggested that *ZNF300* is likely to play an important role in leukemogenesis and hematopoiesis, the exact role of *ZNF300* remains unknown.

In this study, we aimed to reveal the potential role of *ZNF300* in blood cell differentiation by using a K562 cell model. K562 is a human erythroleukemia cell line, approximates to megakaryocyte-erythrocyte progenitor stage, and has the bi-potency to differentiate into megakaryocytes or erythrocytes induced by phorbol-12-myristate-13-acetate (PMA) or cytosine arabinoside (Ara-C), respectively [19]. We demonstrated that *ZNF300* was upregulated in K562 cells undergoing megakaryocytic differentiation induced by PMA or erythrocytic differentiation induced by Ara-C, respectively. Furthermore, *ZNF300* knockdown potently abolished K562 cell differentiation under both conditions. The loss of differentiation capacity in *ZNF300* knockdown cells coincided with increased proliferation evidenced by increased cell percentage at S phase, upregulation of PCNA, and decreased expression of cell cycle regulators p15 and p27. In addition, MAPK/ERK signaling was quenched by *ZNF300* knockdown. These observations suggest that the increased proliferation and impaired MAPK/ERK may contribute to the loss of differentiation capacity in K562 cells.

## Materials and Methods

### Cell culture and differentiation

K562 cells were obtained from the America Type Culture Collection and maintained in RPMI 1640 (GIBCO Life Technologies Inc) containing 10% heat-inactivated fetal bovine serum (GIBCO), 100 Unit/ml penicillin, and 100 **µ**g/ml streptomycin in a humidified chamber with 5% CO_2_ atmosphere at 37°C. For differentiation, K562 cells were induced to undergo megakaryocytic differentiation with 10 nM PMA (Sigma) or induced to undergo erythrocytic differentiation with 1 **µ**M Ara-C (Sigma).

#### shRNA-mediated ZNF300 downregulation

Short hairpin RNA (shRNA) was used to knock down *ZNF300*. The shRNA sequences for targeting *ZNF300* were obtained from the Thermo Open Biosystem website (http://www.thermoscientificbio.com/openbiosystems/) and subjected to BLAST search (http://blast.ncbi.nlm.nih.gov/Blast.cgi) against the NCBI human Non-RefSeq RNA library to ensure that no other gene(s) were targeted. In total, five sequences were chosen to knock down the expression of *ZNF300*. These sequences are 5′-CCTCACAGATTGTGTGACTTT-3′ (shZNF300-1#); 5′-GCCCAATTCTAATCTTGAGAA-3′ (shZNF300-2#); 5′-CCAGATGAATATCAGGCAGAT-3′ (shZNF300-3#); 5′-GCCTTTGCTAAGAAGTCACAA-3′ (shZNF300-4#); 5′-GCCTTCAGTGAGAAGTTTCAT-3′ (shZNF300-5#). Pairs of complementary synthetic oligonucleotides for the *ZNF300* target sequence were annealed together and cloned into pLKO.1 puro vector (http://dharmacon.gelifesciences.com/uploadedfiles/resources/the%20rnai%20consortium%20(trc)%20lentiviral%20shrna%20technical%20manual.pdf) to generate shZNF300 constructs.

To establish stable cell line with *ZNF300* knockdown, we transfected K562 cells with shZNF300 constructs or control vector by electroporation. Briefly, the K562 were washed twice with PBS and resuspended in electroporation buffer at the concentration of 2×10^7^ cells/ml. Four µg of plasmid DNA was mixed with 100 **µ**l of cell suspension. The DNA-cell mixture was subjected to electroporation in a 2 mm cuvette using a Nucleofector II electroporator (Amaxa). The electroporated cells were selected with puromycin (2 **µ**g/ml) for one week. The expression of ZNF300 was measured by western blot analysis and quantitative RT-PCR analysis.

### FACS analysis

Megakaryocytic or erythrocytic differentiation was measured by flow cytometry. Approximately, 1×10^5^ cells were collected and washed with PBS containing 1% BSA and 0.1% sodium azide followed by incubation with PE-conjugated anti-CD61 (GPIIIa) (BD Biosciences) or PE-conjugated anti-CD235a (Biolegend) at 4°C for half an hour. The expression of CD61 and CD235a was measured by flow cytometry on a Beckman CyAn. Data were further analyzed using FlowJo software (Tree Star Inc).

For cell cycle profile analysis, cells were fixed with 2% PFA overnight at 4°C, stained with 1 **µ**g/ml DAPI in the presence of saponin (PBS with 0.1% BSA and 0.05% Saponin) for 2 hrs. The DNA content was measured by flow cytometry. Data were analyzed using ModFit LT (Verity Software House).

### Quantitative RT-PCR analysis

Total RNA was isolated with TRIzol reagent and 1 **µ**g of RNA was used for first-strand cDNA synthesis using RevertAid First Strand cDNA Synthesis kit (Thermo scientific, Lithuania, EU). SYBR Green Bestar Real-time PCR Master Mix (DBI Bioscience) was used and the PCR reactions were run on an ABI 7500 real-time PCR system (Applied Biosystems, Foster City, CA, USA). The PCR amplification conditions were: Denaturation at 95°C for 5 min followed by 95°C 30 sec, 60°C 30 sec, 72°C 30 sec for 40 cycles. Each PCR reaction was performed in triplicates and *GAPDH* was used as an endogenous control for normalization. The relative quantitation of real-time PCR product was measured using the comparative ΔΔC_T_ method [20], [21] and presented as bar graph. Primers used for quantitative RT-PCR are as following: *ZNF300-S* 5′-GGATGTGGCTGTGGATTT-3′, *ZNF300-A* 5′-ATGGCTCTTCTCCTTGTT-3′; *CD41-S* 5-′GATGAGACCCGAAATGTAGGC-3′, *CD41-A* 5′-GTCTTTTCTAGGACGTTCCAGTG-3′; *CD61-S* 5′-GTGACCTGAAGGAGAATCTGC-3′, *CD61-A* 5′-CCGGAGTGCAATCCTCTGG-3′. Primers for γ-Globin and GAPDH were derived from references [13], [22].

### Western blotting analysis

Cell lysates were prepared by lysing cells with RIPA buffer (50 mM Tris-HCl, pH 7.5; 150 mM NaCl; 1% NP-40; 0.25% sodium deoxycholate) supplemented with protease inhibitors (pepstatin, leupeptin, aprotinin, each 10 **µ**g/ml; PMSF, 1 mM) and phosphatase inhibitor (Na_3_Vo_4_, 1 mM). 10 **µ**g of protein was separated by SDS-PAGE and transferred to PVDF membrane. Membranes were blotted with antibodies specific for ERK, phosphorylated ERK, p15, p27, PCNA, ZNF300, or HSC70 at 4°C overnight followed by incubation with appropriate secondary antibodies conjugated with HPR. After extensive wash, membranes were incubated with luminescent substrate (ECL system). The luminescent signal was detected by autography.

### Cell proliferation assay

Cell proliferation assay was performed as previously described [23], [24]. Briefly, 5×10^3^ cells were cultured in triplicates in a 24-well plate. Cells were counted in a hemocytometer everyday. Cell proliferation assay was also performed by using a Cell Counting Kit-8. Briefly, 5×10^3^ cells were seeded in 200 **µ**l culture medium in a 96-well plate in triplicates. On each day, cells were incubated with WST-8 [2-(2-methoxy-4-nitrophenyl)-3-(4-nitrophenyl)-5-(2, 4-disulfophenyl)-2H-tetrazolium] for 2 hours. The absorbance at 450 nm was measured using a microplate reader.

### Wright-Giemsa staining and benzidine staining

Wright-Giemsa staining was performed following the manual from the supplier (Sigma). Cell morphology was observed under a light microscopy. Hemoglobin-containing cells were identified by benzidine staining as described [25]. In brief, cells were collected and washed twice with the cold phosphate-buffered saline and then stained with benzidine solution. Benzidine dihydrochloride (2 mg/ml) was prepared in 0.5 M (3%) acetic acid solution and H_2_O_2_ (1%) was added immediately before use. The cell suspensions were mixed with the benzidine solution in a 1∶1 ratio and incubated for 5 min. Cells with blue-brown-stained cytoplasm were counted as benzidine-staining positive cells and at least 1, 000 cells were counted per sample. The experiments were repeated three times.

### Statistical analysis

All data were the statistics of three independent experiments and presented as mean ± standard deviation. A Student's *t* test (two-tail, unpaired) was used to test the difference in two experiment groups. A *p* value less than 0.05 was considered significance.

## Results

### ZNF300 is upregulated in K562 cells undergoing megakaryocytic differentiation

**Figure 1:**
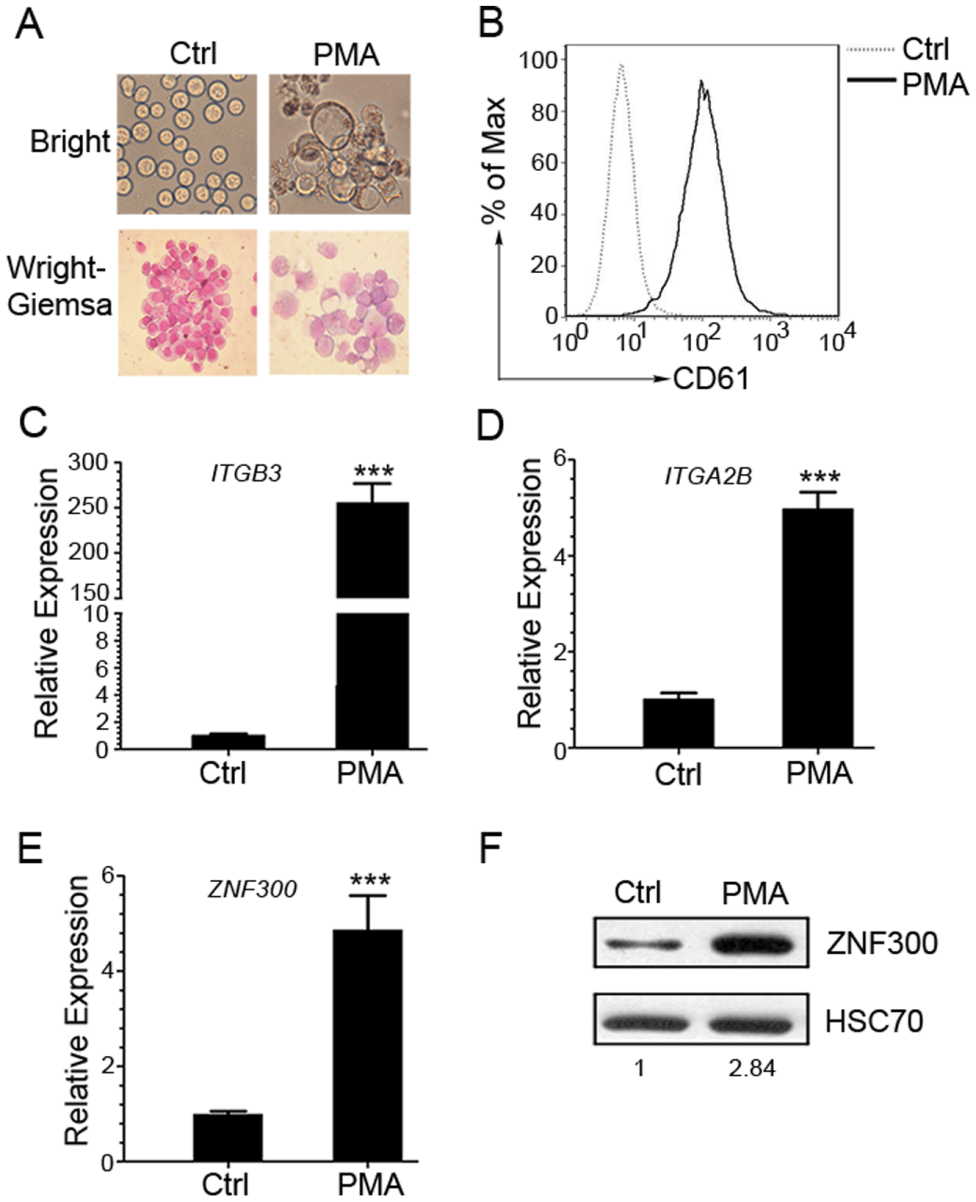
*ZNF300* expression is upregulated in PMA-induced megakaryocytic differentiation in K562 cells. (A) K562 cells were cultured with 10 nM phorbol myristate acetate (PMA) or vehicle control (Ctrl) for 72 hours and stained with Wright-Giemsa stains. The stained or un-stained cells were photographed under microscopy at the bright view of the microscope (magnification ×400 for stained cells and un-stained cells). (B) The resultant cells were also stained with PE-conjugated GPIIIa (CD61)-specific antibody. The samples were analyzed using flow cytometer. Data was analyzed with Flowjo and presented as histogram graph. (C, D) The mRNA level of *ITGB3* (*CD61*) and *ITGA2B* (*CD41α*) in the resultant cells was measured by quantitative RT-PCR. Data was normalized to *GAPDH* and presented as bar graph. (E) The mRNA level of *ZNF300* in the resultant cells was measured by quantitative RT-PCR and represented as the relative expression (mean±SD). Data were representative results of 3 independent experiments with similar results. *** indicates *p*<0.001. (F) The protein expression level of *ZNF300* in resultant cells was measured by western blot and quantified by densitometry. Numbers indicate the densitometry of ZNF300 protein normalized by that of HSC70, which is further normalized to that of untreated cells. Result was the representative blot from 3 experiments with similar result.

Previously, we reported that the ZNF300 protein expression levels correlated to differential stages of leukemic blasts [18]. In addition, *ZNF300* expression was upregulated in HL-60 cells undergoing differentiation induced by DMSO. These results suggest that *ZNF300* likely plays a role in the pathogenesis of leukemia or blood cell differentiation. To address the potential role of *ZNF300* in blood cell differentiation, we chose K562 cells as a model. PMA treatment effectively induced megakaryocytic differentiation in K562 cells. These cells showed typical characters of megakaryocytic differentiation with a marked increase in cell size, extensive multinuclearity, and the presence of vacuoles (**Fig. 1A**). Megakaryocytic differentiation was also evidenced by a significant increase of CD61 (*ITGB3*) expression, the differentiation surface marker of megakaryocytes, determined by flow cytometry and quantitative RT-PCR(**Fig. 1B,C**). The mRNA expression level of CD41 (*ITGA2B*), another differentiation surface marker of megakaryocytes, was also upregulated (**Fig. 1D**). More importantly, PMA treatment also significantly upregulated *ZNF300* expression at both mRNA and protein levels as shown in **Fig. 1E** and **Fig.** **1F** compared to the untreated control. These observations suggest that *ZNF300* upregulation correlate to megakaryocytic differentiation in K562 cells.

### ZNF300 is upregulated in K562 cells undergoing erythrocytic differentiation

**Figure 2:**
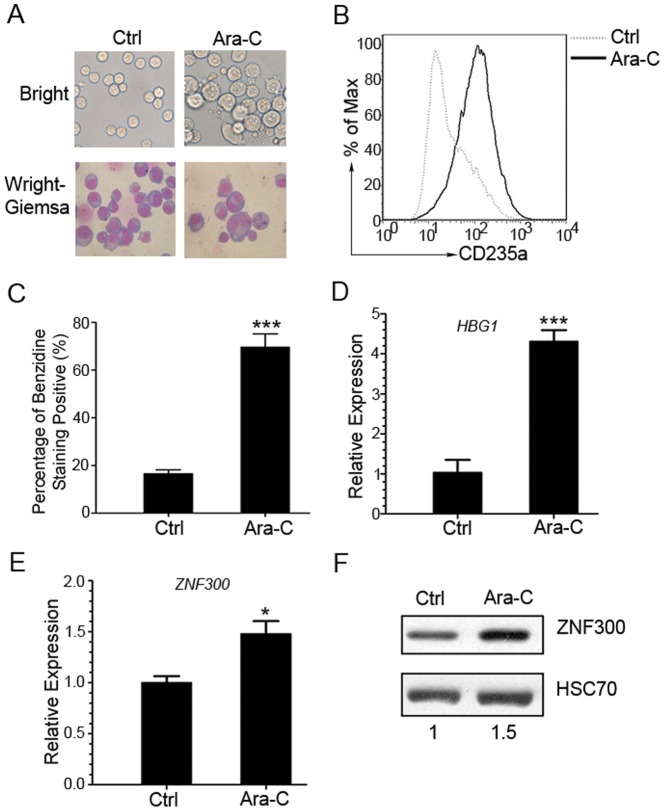
ZNF300 expression is upregulated during the erythrocytic differentiation when K562 cells were induced by Ara-C. (A) K562 cells were cultured in the absence (Ctrl) or presence of 1 **µ**M Ara-C for 168 hours and were stained with Wright-Giemsa stains. Unstained cells were photographed under the dark field and the stained cells were photographed under the bright field (original magnification ×400). (B) The erythrocytic differentiation of resultant cells were determined by staining with PE-conjugated anti-CD235a antibody and analyzed by FACS. Histogram was the representative result from 3 independent experiments with similar results. (C) The erythrocytic differentiation of resultant cells was also determined by benzidine staining to measure the hemoglobin protein. The hemoglobin staining positive cells were counted under light microscope and data were presented as percentage of benzidine staining positive cells. Results were statistics of three independent experiments with similar results (mean±SD). *** indicates *p*<0.001. (D) The mRNA expression level of γ-hemoglobin (*HBG1*) in the resultant cells was measured by quantitative RT-PCR. (E) The mRNA level of ZNF300 in the resultant cells was measured by quantitative RT-PCR and represented as the relative expression (mean±SD). Results were representative data from 3 independent experiments with similar results. *** indicates *p*<0.001. (F) The protein expression level of ZNF300 in resultant cells was measured by western blot and quantified by densitometry. Numbers indicate the densitometry of ZNF300 protein normalized by that of HSC70, which is further normalized to that of untreated cells. Results were the representative blot from 3 experiments with similar results.

To determine whether *ZNF300* expression is altered in K562 cells undergoing erythrocytic differentiation, we treated K562 cells with Ara-C as previously reported [26], [27]. As shown in **Fig. 2A**, the K562 cells treated with Ara-C exhibited condensed nuclei and high proportion of nucleus contraction and fragmentation in contrast to untreated control cells. Erythrocytic differentiation was also evidenced by an increase of CD235a, a differentiation surface maker for erythrocytes, measured by flow cytometry (**Fig. 2B**). In addition, Ara-C treatment also significantly increased the percentage of benzidine-staining positive cells, which measured hemoglobin expression as an endogenous erythrocytic differentiation marker in K562 cells [22], [28] (**Fig. 2C**). The γ-globin expression was confirmed at mRNA level (**Fig. 2D**). Interestingly, we observed upregulation of *ZNF300* at both mRNA and protein levels (**Fig. 2E, F**). These observations suggest that *ZNF300* upregulation correlate to erythrocytic differentiation in K562 cells.

### ZNF300 knockdown abolishes PMA-induced megakaryocytic differentiation and Ara-C-induced erythrocytic differentiation in K562 cells

To establish the causal-effective relationship between upregulation of *ZNF300* and megakaryocyte differentiation, we inhibited *ZNF300* expression in K562 cells by short hairpin RNA (shRNA) technique. We designed 5 different shRNAs and subcloned into pLKO.1 vector to make shRNA-expressing vectors (shZNF300). K562 cells were transfected with shZNF300 or control constructs (Ctrl) and selected with puromycin. As shown in S1 Figure, three out of 5 constructs (shZNF300 #2, 3, 4) significantly knocked down *ZNF300* expression at both mRNA and protein levels. In addition, *ZNF300* knockdown did not significantly alter CD61 or CD235a expression determined by flow cytometry at basal level (data not shown). Thus three cell lines derived from K562 cells stably transfected with three shZNF300 constructs (shZNF300#2, #3, #4) were used for further experiments.

**Figure 3:**
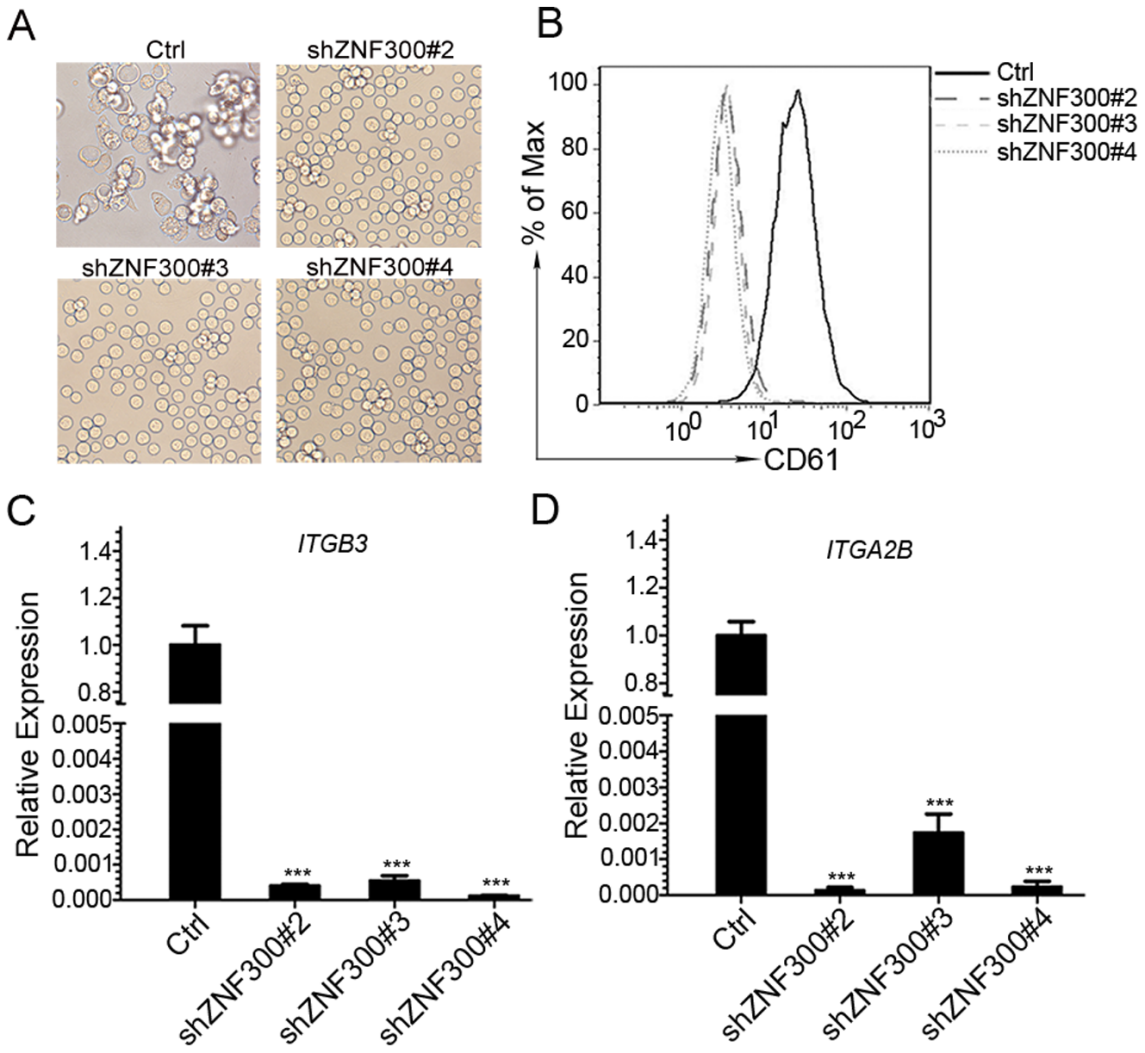
*ZNF300* knockdown abolished megakaryocytic differentiation. (A) Control and *ZNF300* knockdown (shZNF300) cells were cultured in the presence of 10 nM PMA for 72 hours. The morphology of the treated cells was observed under the light microscope (×400 magnifications). (B) The megakaryocytic differentiation of the treated cells was measured by staining cells with PE-conjugated anti-CD61 antibody and analyzed by FACS. (C) The megakaryocyte differentiation of the treated cells was measured by detecting *ITGB3* mRNA level (quantitative RT-PCR) and presented as relative expression level. (D) The megakaryocytic differentiation of the treated cells was also measured by detecting *ITGA2B* mRNA level (quantitative RT-PCR) and presented as relative expression level. Data were representatively results of 3 independent experiments with triplicates. *** indicates p<0.001

To examine the effect of *ZNF300* knockdown on megakaryocytic differentiation in K562 cells, control (Ctrl) or *ZNF300* knockdown cells (shZNF300) were treated with PMA. As previously described, control cells exhibited typical characters of megakaryocytic differentiation after PMA induction. In contrast, shZNF300 cells appeared to be insensitive to PMA treatment: these cells looked bright and remained in round shape, suggesting that *ZNF300* knockdown diminishes megakaryocyte differentiation (**Fig. 3A**). Indeed, PMA treatment failed to upregulate CD61 (*ITGB3*) expression in shZNF300 cells (shZNF300#2, 3, 4) compared to control cells (Ctrl) (**Fig. 3B, C**). Upregulation of CD41 (*ITGA2B*) mRNA expression was also eliminated by *ZNF300* knockdown (**Fig. 3D**). Consistently, the shRNAs that failed to efficiently knock down ZNF300 (shZNF300#1 and 5) lost the ability to abrogate megakaryocyte differentiation (data not shown). In addition, PMA also failed to promote megakaryocytic differentiation in HEL cells with ZNF300 knockdown (data not shown). These results suggest that *ZNF300* knockdown abrogate megakaryocyte differentiation induced by PMA treatment.

**Figure 4:**
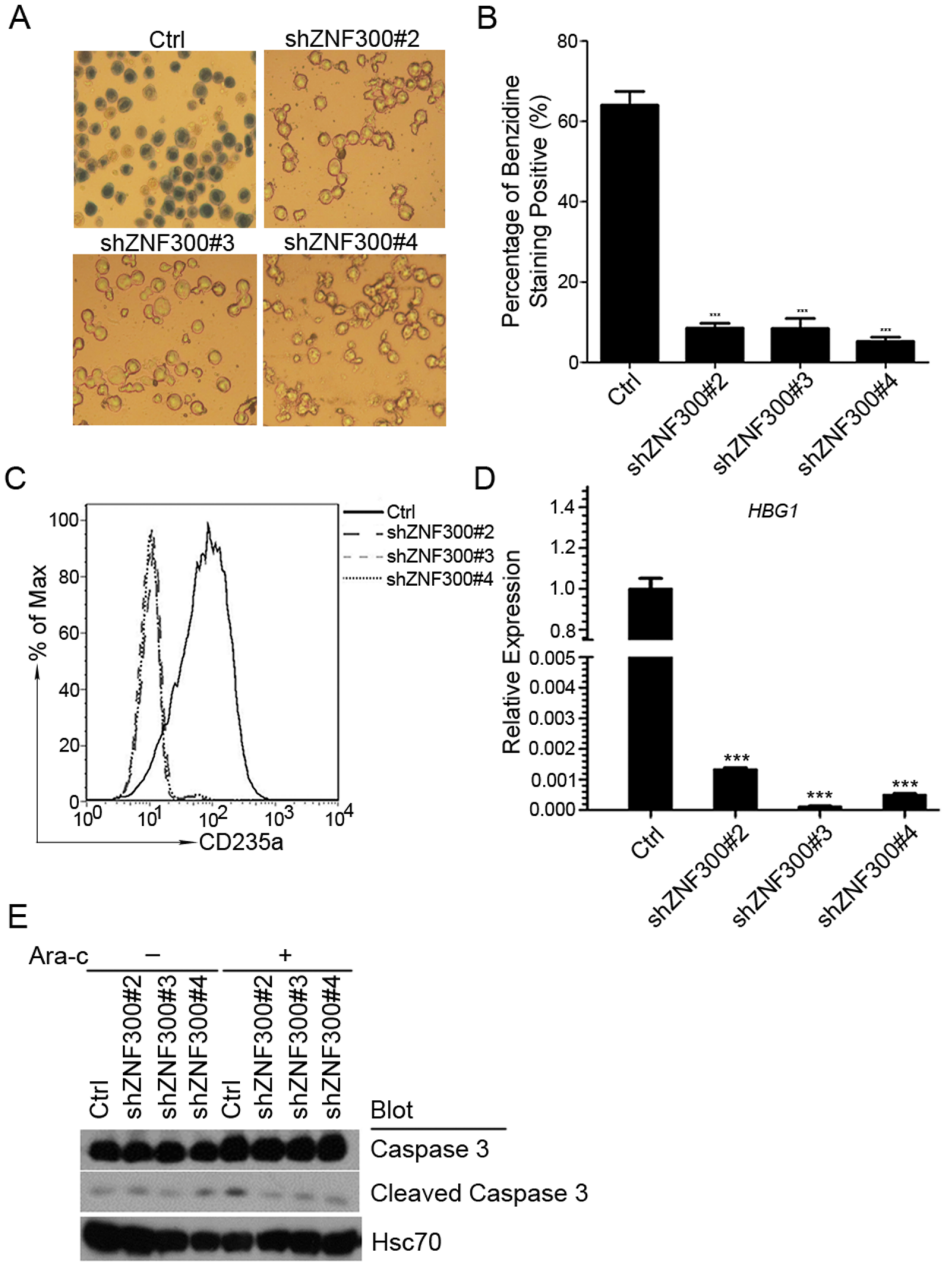
*ZNF300* knockdown blocks Ara-C-induced erythrocytic differentiation. (A) Control and *ZNF300* knockdown cells (shZNF300) were cultured in the presence of Ara-C for 72 hours. The resultant cells were stained with benzidine to measure the hemoglobin protein. The stained cells were photographed under the bright field (original magnification ×200). (B) The hemoglobin staining positive cells were counted under microscope and data were presented as percentage of benzidine staining positive cells. The bar graph was the statistics of benzidine staining. (C) The erythrocyte differentiation of resultant cells was determined by staining cells with PE-conjugated CD235a antibody and measured by FACS. (D) The erythrocyte differentiation of resultant cells was also determined by detecting mRNA level of *γ-hemoglobin* (*HBG1*) through quantitative RT-PCR. *** indicates *p*<0.001. (E) Control and *ZNF300* knockdown cells treated with (+) or without (−) Ara-C were collected for western blot with antibodies as indicated.

To assess the role of *ZNF300* in erythrocytic differentiation induced by Ara-C, control (Ctrl) or *ZNF300* knockdown cells (shZNF300) were treated with Ara-C. As shown in **Fig. 4A**, Ara-C treatment led to high percentage of benzidine-staining positive cells in control cells (Ctrl). In contrast, benzidine-staining positive cells in *ZNF300* knockdown cells (shZNF300) were barely observed (**Fig. 4A, B**), suggesting that *ZNF300* knockdown abrogates erythrocytic differentiation induced by Ara-C. The diminished erythrocytic differentiation in shZNF300 cells was also confirmed by failure to upregulate CD235a and γ-globin expression compared to that of control (Ctrl) (**Fig. 4C, D**). In addition, we measured the cleaved caspase 3 (**Fig. 4E**). As expected, we barely detected any cleaved caspase 3 in control cells (Ctrl) or ZNF300 knockdown cells without Ara-C treatment unless we overexposed the film as shown in **Fig. 4E**. With Ara-C treatment, only slight upregulation of cleaved caspase 3 was observed in control cells but not in ZNF300 knockdown cells. These results were consistent to previous reports showing that Ara-C treatment did not induce significant apoptosis [2], [29]. These observations suggest that *ZNF300* knockdown block erythrocytic differentiation induced by Ara-C without affecting apoptosis.

### ZNF300 knockdown promotes cell proliferation in K562 cells

**Figure 5:**
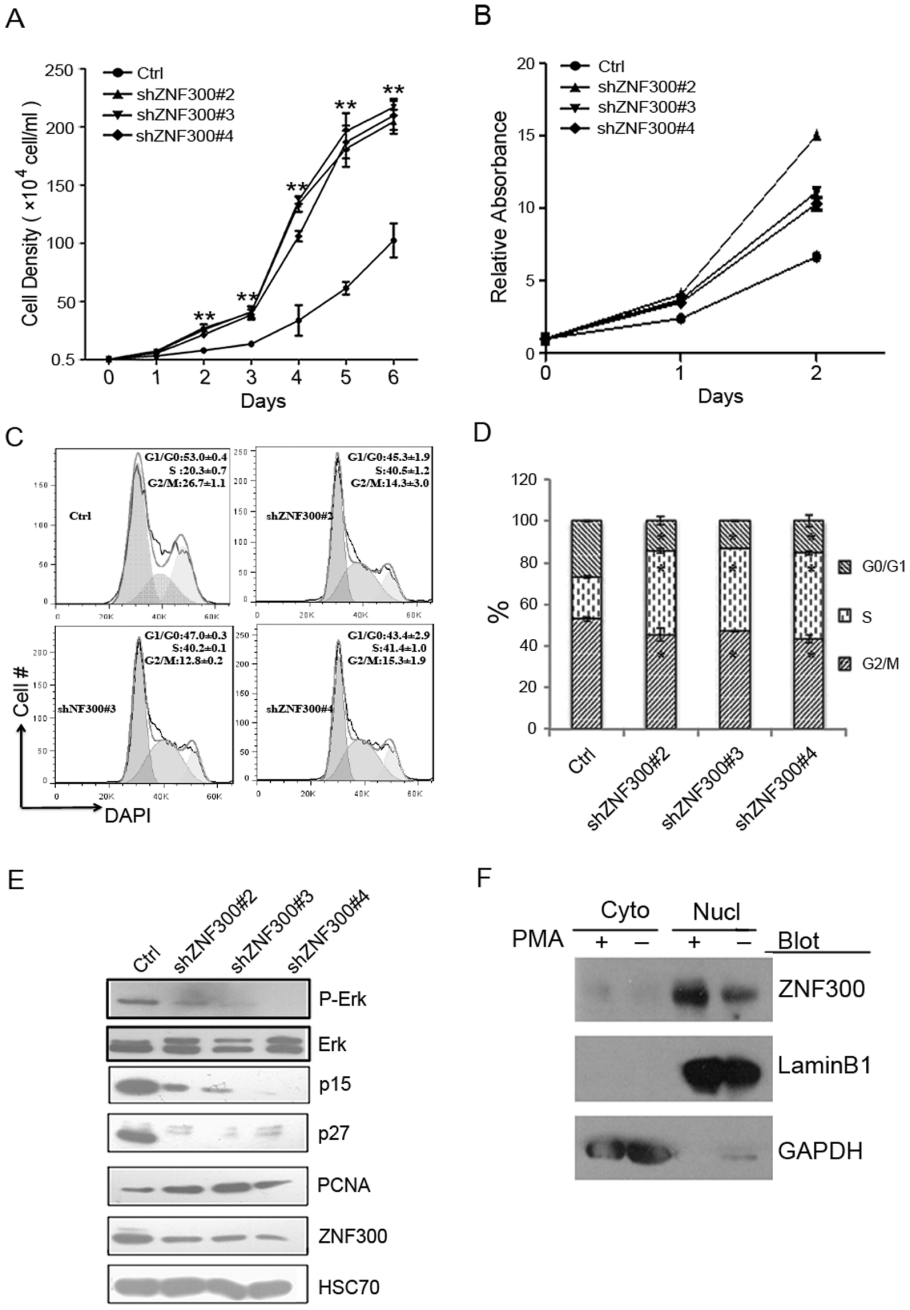
*ZNF300* knockdown promotes proliferation in K562 cells. (A) The same amount of control and *ZNF300* knockdown cells (5×10^3^) were plated in triplicates in a 24-well plate and the cell number was counted for consecutive 6 days. Data were statistics (mean ±SD) of representative results from 3 independent experiments with similar results. (B) Cell proliferation assay was also performed by using Cell Counting Kit-8. The absorbance at 450 nm was measured for consecutive 3 days and normalized to that of the first day. The cell proliferation was presented as relative absorbance. (C) Control and *ZNF300* knockdown cells were fixed, permeablized, and stained with DAPI. The DNA content was analyzed by FACS. The distribution of cells in G0/G1, S, and G2/M phases was further analyzed by ModFit LT. Data were the statistics of representative results from 3 independent experiments with similar results. Numbers indicate the percentage (mean ±SD). (D) Bar graph of the statistics of cell cycle profiling experiments. (E) Cell lysates were prepared from control or *ZNF300* knockdown cells and the protein expression level was detected by western blot with antibodies as indicated. HSC70 served as a protein loading control. ** indicates *p*<0.01. (F) The cytosol fraction (Cyto) and nucleus (Nucl) fraction of K562 cells treated with (+) or without (−) PMA were used for western blot with antibodies as indicated.

Failure to undergo differentiation frequently accompanies increased proliferation in blood cells. Thus we investigated the effect of ZNF300 knockdown on cell proliferation. We measured cell proliferation by two means. One was to count viable cells and the other was to detect dehydrogenase activity with CCK-8. In two days, the number of viable shZNF300 cells (shZNF300#2, 3, 4) significantly exceeded that of control cells (Ctrl) and the discrepancy was dramatically amplified over time (**Fig. 5A**). Consistently, the relative absorbance of ZNF300 knockdown cells (shZNF300#2, 3, 4) was higher than that of control cells (Ctrl) (**Fig. 5B**). In contrast, cells stably transfected with shZNF300#1 and 5 that failed to knock down ZNF300 proliferated normally comparable to that of control cells (data not shown). These observations suggest that ZNF300 knockdown promote cell proliferation in K562 cells. To support this, cell cycle profile analysis demonstrated that shZNF300 cells exhibited increased percentage of cells at S phase. As shown in **Fig. 5C and 5D**, the percentage of cells at S phase in shZNF300 cells were 40.5%, 40.2%, and 41.4% respectively compared to 20.3% in control cells and the difference was significant. Consistently, cell cycle regulator p15 and p27 was downregulated in shZNF300 cells and the proliferation marker PCNA was upregulated (**Fig. 5E**). These results suggest that ZNF300 somehow affect cell cycle progress and ZNF300 downregulation lead to increased proliferation.

Sustained MAPK/ERK signaling is essential for megakaryocyte differentiation in K562 cells. We thus examined the phosphorylation of ERK in *ZNF300* knockdown cells. We found that the phosphorylation of ERK (P-ERK) was significantly reduced in *ZNF300* knockdown cells compared to that in control cells (**Fig. 5E**). This result was consistent to the phenotype that shZNF300 failed to undergo megakaryocytic differentiation.

Previously, ZNF300 was shown to localize in both cytosol and nucleus [30]. To test whether alteration of ZNF300 subcellular distribution may contribute to the phenotype, we measured the protein level of ZNF300 in both cytosol and nucleus. We found that ZNF300 dominantly localized in cytosol (Cyto) and PMA treatment did not alter the distribution (**Fig. 5F**).

Taken together, the increased proliferation and impaired MAPK/ERK signaling may contribute to the effect of *ZNF300* knockdown on proliferation and differentiation in K562 cells.

## Discussion

Previously, *ZNF300* was shown to correlate with Crohn's disease and 5q-syndrome [16], [31]. Further studies suggest that *ZNF300* may play a role in cell proliferation, apoptosis and immune response [12], [13]. In this study, we discovered that *ZNF300* downregulation abolished forced differentiation in K562 in response to PMA or Ara-C treatment. Our study suggests a novel function of *ZNF300* in megakaryocytic and erythrocytic differentiation.

*ZNF300* function study has been impeded in part due to its lack of orthologous in mice. In order to study its function, we tried to overexpress *ZNF300* in K562 by lentiviral transduction. We failed to obtain any transductants that stably expressed full length *ZNF300* (data not shown). This is similar to another research on *ZNF268* showing that no transfectants expressing full length *ZNF268* could be established in HEK293 cells [32]. Thus knockdown of *ZNF300* is the only choice. These observations suggest that KRA-ZFPs may play important roles and have to be tightly regulated. However, how KRAB-ZFPs are regulated is largely unknown. Recent ChIP-Seq data of KRAB-associated protein 1 (KAP1), the most important partner of KRA-ZFPs, showed that KAP1-binding was significantly enriched in the zinc finger region of KRAB-ZFPs [33]. These observations suggest that KRAB-ZFPs may negatively regulate themselves and mediate long-range heterochromatinization. This may partially explain the reason why *ZNF300* could not be overexpressed. Further study on the regulation of *ZNF300* will significantly help us understand how *ZNF300* exerts its function.

ZNF300 may play multiple functions as transcription factor and signaling molecule. As a typical KRAB-ZFPs, ZNF300 protein bears 12 zinc finger motifs. Interestingly, ZNF300 localizes in both cytoplasm and nucleus [34]. In HeLa cells, ZNF300 enhanced NF-κB signaling and promoted tumorigenesis in a xenograft nude mice model [14]. In contrast, *ZNF300* knockdown promoted cell proliferation in K562 cells in this study. We speculate that two possibilities may explain the apparent inconsistency. On one hand, the same signaling molecule affected by ZNF300 may play completely opposite functions in different cell types. For instance, MAPK/ERK signaling is activated in various types of carcinoma and supposed to be one of critical signaling pathways for carcinogenesis [35], [36]. However, MAPK/ERK is critical for megakaryocyte differentiation in K562 cells. Therefore, the impaired MAPK/ERK may explain the failure to undergo megakaryocyte differentiation in *ZNF300* knockdown cells. Comparison of signaling pathway affected by *ZNF300* in carcinoma cells and leukemic cells may provide more information. On the other hand, the target genes regulated by *ZNF300* may be different in these cells. Although the potential *ZNF300* DNA-binding consensus sequence was determined, very few target genes were identified. Further study using microarray or ChIP sequencing may significantly promote study on *ZNF300* function.

The increased proliferation may contribute to impaired differentiation phenotype in *ZNF300* knockdown cells. ZNF300 knockdown cells showed impaired erythrocytic differentiation by Ara-C and increased proliferation (**Fig. 2** and **Fig. 5**). Our findings supported a previous study showing that hemoglobin induction by Ara-C in K562 cells was cell-cycle dependent [26]. Our study also support a previous report showing that nuclear receptor co-repressor N-CoR was required for Ara-C-induced erythrocyte differentiation in K562 cells using similar knockdown technique [37]. However, N-CoR seemed not to be required for PMA-induced megakaryocytic differentiation of K562 cells. Given that both KRAB-ZFPs and N-CoR can mediate gene repression at chromosomal level, it is possible that change in chromatin structure may be a common feature of erythrocyte differentiation. Indeed, a recent work showed that developmentally silenced globin genes could be reactivated by forced chromatin looping [38]. Further study on this may facilitate us to understand the erythrocyte differentiation at chromosomal level.

Taken together, we have discovered that *ZNF300* may control expression of cell cycle regulators as well as signal transduction in K562 cells that subsequently lead to increased proliferation and resistance to PMA- or Ara-C-induced cell differentiation.

## Supporting Information

S1 Figure **ZNF300 downregulation by shRNAs.** The K562 cells were transfected with control vector (Ctrl) or vector expressing shRNA specific for human ZNF300 (shZNF300). The expression of ZNF300 was measured by quantitative RT-PCR (A) or Western Blot (B).
(TIF)
