# Supplementary figures and images for: ZNF300 Knockdown Inhibits Forced Megakaryocytic Differentiation by Phorbol and Erythrocytic Differentiation by Arabinofuranosyl Cytidine in K562 Cells
Source: PLoS One. 2014 Dec 8;9(12):e114768. doi: 10.1371/journal.pone.0114768 (PMC4259388; doi:10.1371/journal.pone.0114768)

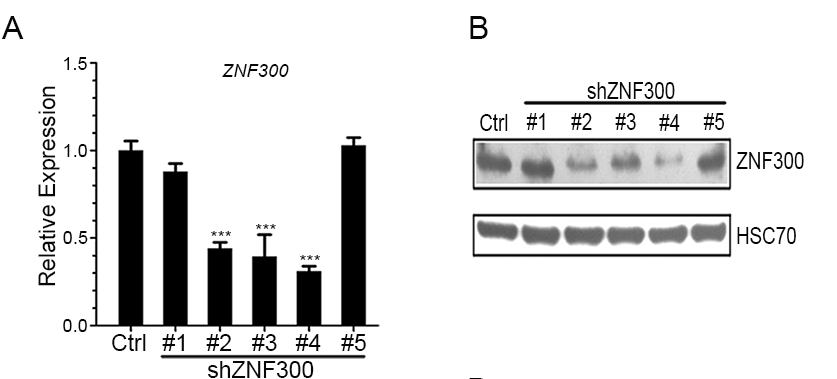

Supplement: S1 Figure — ZNF300 downregulation by shRNAs. The K562 cells were transfected with control vector (Ctrl) or vector expressing shRNA specific for human ZNF300 (shZNF300). The expression of ZNF300 was measured by quantitative RT-PCR (A) or Western Blot (B). (TIF) [file pone.0114768.s001.tif]
